# An Insight into Functional Metagenomics: A High-Throughput Approach to Decipher Food–Microbiota–Host Interactions in the Human Gut

**DOI:** 10.3390/ijms242417630

**Published:** 2023-12-18

**Authors:** Elliot Mathieu, Véronique Léjard, Chaima Ezzine, Pauline Govindin, Aurélien Morat, Margot Giat, Nicolas Lapaque, Joël Doré, Hervé M. Blottière

**Affiliations:** 1 Université Paris-Saclay, INRAE, MGP Metagenopolis, 78350 Jouy-en-Josas, France; elliot.mathieu@inrae.fr (E.M.); veronique.lejard@inrae.fr (V.L.); chaima.ezzine@inrae.fr (C.E.); pauline.govindin@inrae.fr (P.G.); aurelien.morat@inrae.fr (A.M.); margot.giat@inrae.fr (M.G.); joel.dore@inrae.fr (J.D.); 2 Université Paris-Saclay, INRAE, AgroParisTech, Micalis Institute, 78350 Jouy-en-Josas, France; nicolas.lapaque@inrae.fr; 3 Nantes Université, INRAE, UMR 1280, PhAN, 44000 Nantes, France

**Keywords:** microbiome, gut microbiota, metagenomics, bacteria–host crosstalk

## Abstract

Our understanding of the symbiotic relationship between the microbiota and its host has constantly evolved since our understanding that the “self” was not only defined by our genetic patrimony but also by the genomes of bugs living in us. The first culture-based methods highlighted the important functions of the microbiota. However, these methods had strong limitations and did not allow for a full understanding of the complex relationships that occur at the interface between the microbiota and the host. The recent development of metagenomic approaches has been a groundbreaking step towards this understanding. Its use has provided new insights and perspectives. In the present chapter, we will describe the advances of functional metagenomics to decipher food–microbiota and host–microbiota interactions. This powerful high-throughput approach allows for the assessment of the microbiota as a whole (including non-cultured bacteria) and enabled the discovery of new signaling pathways and functions involved in the crosstalk between food, the gut microbiota and its host. We will present the pipeline and highlight the most important studies that helped to develop the field. To conclude, we will emphasize the most recent developments and hot topics in functional metagenomics.

## 1. Metagenomics of the Human Microbiota

In recent decades, the perception of the human body has been challenged by an awareness of microbiota. The billions of microorganisms that colonize our body are, nowadays, not seen as insignificant or pathogenic. The intestinal microbiota has received the most attention by far and has been shown to contribute to our energy supply via the fermentation of non-digestible residues by our digestive enzymes. Fibers are a great example of residues that could not used by our bodies without the support of the gut microbiota [1]. More recently, a considerable number of studies have highlighted the role of the intestinal microbiota in the development and maturation of the immune system, its importance in the control of metabolic functions, its interaction with the central nervous system, and its contribution to protecting against colonization by pathogens (also called the barrier effect) [2,3,4,5,6,7]. Other body parts have also been investigated in terms of their microbiota, and these studies keep tackling the concept of the human “self”. A hallmark example is the recent discovery of the lung microbiota [8]. This organ was considered sterile until the early 21st century, and the presence of microorganisms was only considered indicative of pathological conditions. Like the intestinal microbiota, the microbiota of the lungs, skin, vagina, and the oral and nasal cavities were shown to be important in human physiology [9,10,11,12]. The new perception of the human body that we refer to is the concept of a “holobionte”, which designates the whole constituted by a host and its microbial inhabitants. 

The microbiota colonizes the human body from birth and will diversify, depending on the body parts, over the first months (e.g., the first two months for the lung microbiota) and first years of life (e.g., the first three years for the intestinal microbiota) [13,14,15]. The diversification of the microbiota occurs in parallel with the development and maturation of the immune system. This microbial colonization is influenced by diverse factors such as the mode of birth (natural delivery versus caesarean section), the mode of feeding (breastfeeding versus formula feeding), environmental hygiene, the intake of drugs, including antibiotics, and the mode and time of weaning [7,14]. At adult age, for a healthy person, it is considered that the intestinal microbiota is relatively stable over time. However, the modification of our nutritional habits and several pathologies have been shown to modify the composition and functions of the gut microbiota [16]. 

Studies on the composition and functions of microbiota imply the use of several techniques, such as microbiology, molecular biology, microbial ecology and immunology. Before the recent development of next-generation sequencing, molecular approaches (e.g., targeting the 16S ribosomal deoxyribonucleic acid (DNA)) allowed us to understand the complexity of the microbiota and highlight the importance of its non-cultivable fraction. Indeed, since the first studies on the microbiota, and for a long time, the characterization of the microbiota has been limited to the study of cultivable microorganisms. Therefore, our understanding was limited to a narrow field of vision. Recent advances in next-generation sequencing techniques and metagenomic approaches have permitted major advances in the understanding of the microbiota. 

The first step of metagenomics is to collect samples, such as human feces or saliva. Major attention must be paid to this step in order to perform optimal microbiome analysis [17]. To do so, operational standardized procedures were designed and provided to the scientific community [18]. The study of the intestinal microbiota has been facilitated by the analysis of feces and by its density and abundance, but other microbiota, such as in the lungs, require more invasive sampling and advanced techniques. From samples of interest, two strategies are available to sequence the DNA: a targeted approach, i.e., the amplification of a gene via polymerase chain reaction and then the sequencing of all the amplicons obtained, and a more global approach, i.e., the shotgun metagenomic method that involves the direct sequencing of the entire DNA presents in the samples without any target amplification [17]. Shotgun metagenomics consists of generating a high number of short sequences, or reads, of 100 to 150 base pairs (bp) that will further be reported to a catalogue of known genes in order to identify and quantify those present in the sample [19,20]. To date, the most complete catalogue contains 10.2 million genes. Quantitative metagenomic studies enabled the characterization of the composition of the intestinal microbiota and indicated that no common microbiota could be observed in healthy individuals. Indeed, the MetaHIT project showed that at least three enterotypes could be observed [21]. These enterotypes are dominated by different bacterial genera. Nutritional habits and the host’s physiology are essential determinants of these enterotypes. The richness in bacterial genes is also a key characteristic of the gut microbiota. Not all individuals are equal in regard to their bacterial gene richness, ranging from less than 300,000 to more than 800,000 genes [22]. Aside from the analysis of healthy individuals, metagenomic studies have allowed the analysis of the gut microbiota in different pathological contexts and have proven to be great prognosis and diagnostic tools. Indeed, quantitative metagenomic studies highlighted dysbiosis and a loss of richness in patients with Crohn’s disease, obesity or hepatic cirrhosis [17,19,23,24]. The analysis of the presence or absence of several species in feces samples constitutes a potentially simple and non-invasive diagnosis for inflammation of the digestive tract or liver damage. In addition, quantitative metagenomics can provide information about microbiota function through gene analysis. For example, the analysis of overrepresented genes in cirrhosis patients highlights an increase in genes related to the production of ammoniac and gamma amino-butyric acid, factors associated with the exacerbation of cirrhosis [25]. While giving tremendous amounts of information, quantitative metagenomics does not provide insights into cellular mechanisms and molecular interactions between the host and its microbiota. To date, our knowledge of these interactions remains patchy. In the following sub-chapters, we will describe how the functional metagenomics approach is relevant to deciphering microbiota-host interactions but also food–microbiota interactions.

## 2. Functional Metagenomics of Food–Microbiota–Host Interactions

The relationship between the gut microbiota and the host is the result of a long co-evolution and adaptations that benefit both parties. The host provides the ecological niche and the nutrients needed for bacterial development, and the microbiota produces essential metabolites for humans, such as certain vitamins or short-chain fatty acids that contribute to the physiological functions of the host [26,27]. The microbiota is involved in intestinal homeostasis and immune system maturation [2,3]. Like investigations into microbiota composition, the exploration of the complex relationships between the microbiota, food, and the host was limited to culture-based research. Recently, functional metagenomics has proven to be a powerful technique for the discovery of new signaling pathways and functions in the crosstalk between nutrients, the gut microbiota and the host. First, we will describe the basic principle of this approach, then the use of functional metagenomics to study food–microbiota and microbiota–host interactions, and we will conclude with the very recent advances in functional metagenomics. 

### 2.1. Functional Metagenomic—The Pipeline Explained

**Figure 1:**
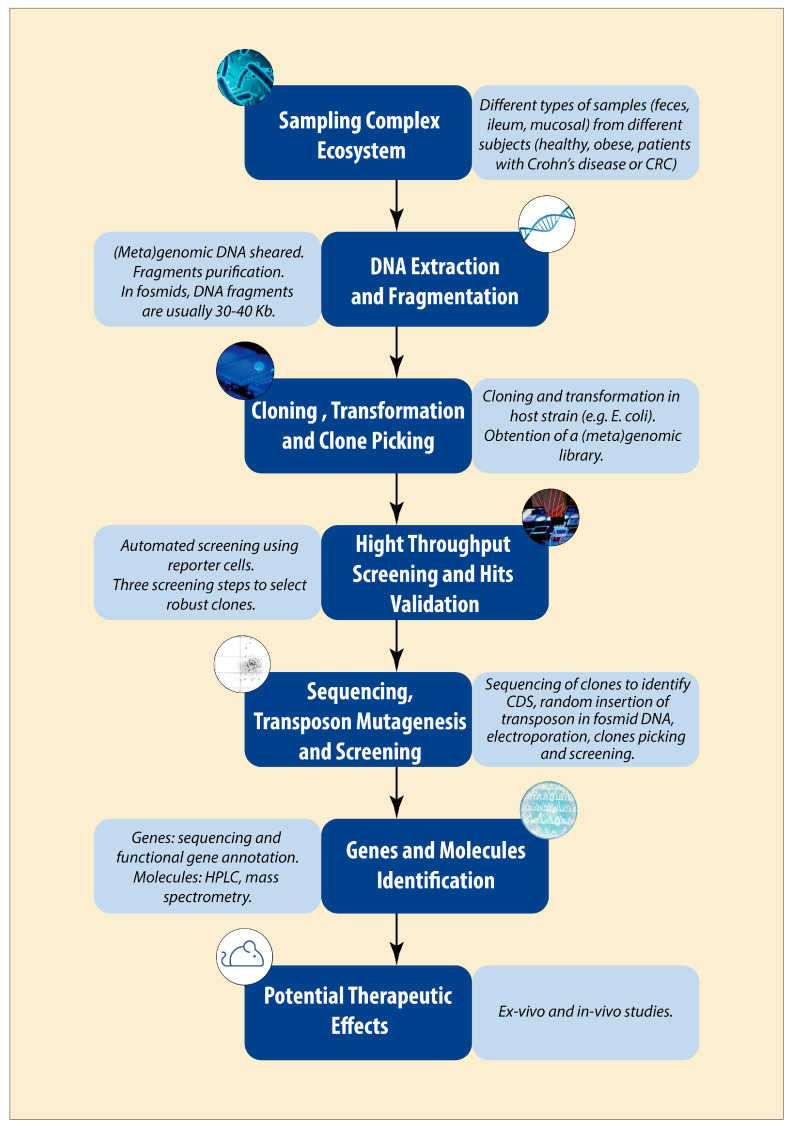
Schemes of the functional metagenomic pipeline developed at MetaGenoPolis. CRC: colorectal cancer; *E. coli*: *Escherichia coli*; DNA: deoxyribonucleic acid; CDS: coding sequence; HPLC: high-performance liquid chromatography.

Before delving into the specifics of using functional metagenomics to unravel food–microbiota–host interactions, it is important to understand the basic principle of this approach. The first step consists of sampling an ecosystem of interest, the gut microbiota, for example. As previously mentioned, for quantitative metagenomics, great attention must be paid to this point in order to perform optimal analysis. Indeed, keeping the bacterial DNA as intact and as representative of its composition as possible is necessary. As shown in Figure 1, the next stage consists of extracting the DNA derived from the bacterial genome of the sample, which is called metagenomic DNA, and shearing it into fragments. Usually, metagenomic DNA is sheared into large fragments of 10 to 50 kb, which give access to full operon and operational gene clusters. After purification, these fragments are ligated into a vector, i.e., a plasmid, cosmid, or fosmid, before their transformation into a host bacterium that accepts only one copy. *Escherichia coli* (*E. coli*) is the major species used as a host strain in most metagenomic studies. *E. coli* makes a suitable host due to its ease of cultivation and fast and efficient growth, but also as the expression of heterologous genes allows access to about 40% of the genes for both Gram-negative and Gram-positive bacteria [28]. Note that this number is a prediction and englobe the genes from archaea. Strong variations are observed between different groups of organisms (ranging from 7 to 73% (for the *Firmicutes*)). A study by Handelsman et al. showed that more than 50% of the traits of *Bacillus cereus* were expressed in *E. coli* [29]. However, the intestinal microbiota is mainly composed of Gram-positive bacteria, and efforts are thus made to develop systems to use Gram-positive bacteria as hosts. The use of host Gram + bacteria gives access to signals and functions specific to Gram-positive bacteria that enhance the heterologous expression of genes for this type of bacteria. The recent advances regarding the development of Gram-positive bacteria as host cells will be developed in part 2.d (future advances in functional metagenomics). After *E. coli* transformation or infection, single-colony picking is performed in order to obtain a library of metagenomic clones that is composed of a few thousand to tens of thousands of clones. Each of these clones harbors a metagenomic insert, whose size depends on the studies. Once the library of metagenomic clones is produced, a screening step is performed depending on the activity of interest. Different types of screening were developed to search for the bacterial genes involved in the modulation of cell-signaling pathways in reporter human cells or in the hydrolysis of substrates of interest. Regarding the high number of clones generated, the screening is performed in a high-throughput manner. The automation was facilitated by measuring easily readable activities through absorbance, fluorescence, or luminescence in the reporter cells. The positive metagenomic clones obtained, i.e., with interesting characteristics, are sequenced in order to identify the sequences and from which bacteria the metagenomic insert was derived. At this step, the genes that bear the activity are not precisely identified, i.e., it can be due to one or several genes from the initial metagenomic insert. It is necessary to perform transposon mutagenesis that leads to the creation of hundreds of subclones, which will then be screened for their activity. The mutant clones that have lost the activity are further sequenced, and the genes of interest are identified through comparison with the normal sequences. The clones that bear the genes of interest can be subsequently analyzed in order to identify the produced molecules and to determine their mechanism of action. Their potential therapeutic effects can also be tested via ex vivo or in vivo experiments.

Herein, we explain the functional metagenomics pipeline, from the metagenomics DNA to clones of interest that harbor potential active genes. It is important to note that this workflow can be performed on the genomic DNA of a single bacterial strain. In that case, we talk about functional genomics. In the following parts, we will describe how this approach is employed to decipher the food–microbiota–host interactions and which screening systems are used to discover new interaction mechanisms and molecules of interest.

### 2.2. Functional Metagenomics of Food–Microbiota Interactions

Humans are omnivores and consume a broad range of food, including fruits, vegetables, legumes, nuts, grains, dairy, meats, and fish, to mention a few. These foods are composed of different dietary constituents, such as proteins, sugars, saturated and unsaturated fats, salts, oligosaccharides, and polysaccharides, which are directly or indirectly digested by the human body. The long co-evolution and adaptation of the host and its microbiota have benefited both parties. Indeed, while the microbiota benefits from the ingested nutrients for bacterial development, the host relies entirely on the intestinal microorganisms to be able to use certain dietary constituents. As we will see, the development of culture-independent techniques, especially functional metagenomics, allowed us to unravel important functions of the intestinal microbiota as a whole (i.e., the cultivable and uncultivable fractions of the intestinal microbiota). 

In 2005, Walter and colleagues successfully demonstrated the use of functional metagenomics to investigate the metabolic structure of the mice bowel community and to detect *β*-glucanase enzymes encoded by the metagenomes of the gut microbiota [30]. By using *E. coli* as a host, they generated a metagenomic library of 5760 clones, each clone bearing, on average, a 55 kb insert, representing 320 Mb of metagenomic DNA. To detect clones with *β*-glucanase activity, they screened the library for the expression and secretion of *β*-glucanase that degraded lichenin (lichenin agar plates and Congo red solution). Out of the metagenomic library, they identified three clones that encode proteins with similarity to known *β*-glucanase and four novel *β*-glucanases. Interestingly, the analysis of the sequences present in the clones also showed genomic properties that reflected an adaptation of the microorganisms to the bowel ecosystem. It is also important to note that this study allowed a very limited coverage of the metagenome due to the small size of the library. Limited coverage of the metagenome was also observed in the works of Ferrer et al., which screened only 7% of a metagenomic library originating from the rumen content of a dairy cow [31]. They produced a library of 14,000 clones, each clone harboring an 8 kb insert, representing 77 Mb of metagenomic DNA. The library was screened on agar plates containing α-naphthyl acetate, ostazin brilliant red-hydroxy-ethyl cellulose or *β*-cyclodextrin to identify clones with esterase, cellulase, and amylase-like activities. They identified and characterized 22 novel enzymes with hydrolytic activities. These identified enzymes originated from 19 clones. Despite the limited coverage of the metagenome, both studies highlighted the relevance of the functional approach by using a large insert library. 

Later, the use of functional metagenomics was successfully undertaken by Jones and colleagues in 2008 to analyze the bile salt hydrolase activity of the human gut microbiota [32]. Bile salts are not dietary compounds but are derived from lipid metabolism and act as signaling molecules that regulate their own biosynthesis, cholesterol homeostasis, and local mucosal defenses in the intestine. In this study, they screened 89,856 fosmid clones originating from a metagenomic library that was previously constructed from a human fecal sample [33]. Compared to the two previous studies described, the bank of clones represented 3.6 Gb of metagenomic DNA, which is equivalent to the dominant intestinal metagenome. Therefore, this provided a more robust representation of the ecosystem of interest. They screened the clones for their bile salt hydrolase activity using a bile agar plate supplemented with glycol-conjugated bile acid (CBA), tauro-CBA, and human bile. Out of the 89,856 clones, they obtained 142 positive clones from which the sequences of 90 clones were affiliated to the members *Firmicutes*, *Bacteroidetes*, and *Actinobacteria*. The remaining clones were affiliated with potential novel and uncultured members of the human gut microbiota. Interestingly, clones affiliated to *Firmicutes* and *Actinobacteria* were capable of degrading glyco-CBA, tauro-CBA, and human bile, while the clones affiliated with *Bacteroidetes* were generally only able to hydrolyze tauro-CBA. Jones et al. [33] demonstrated the powerful capacity of the functional metagenomic method to discover the functions of the human gut microbiota. Apart from their role in bile salt hydrolase activity, the bacteria of the human gut are involved in the adaptation to the induction of diet stress. Specifically, the ability of gut bacteria to adapt to changes in osmolarity is a major determinant of their survival. In 2012, the same laboratory used functional metagenomics to explore the salt tolerance of the human gut microbiota [34]. In this study, they used the same metagenomic library [32,33] and screened 23,040 clones, each bearing inserts of about 40 kb, on an agar plate containing 6.5% NaCl. They identified 53 positive clones, from which 6 grew within 24 h on plates, while the other 47 grew within 36 h. All the positive clones were then sequenced, and the analysis taxonomically assigned the sequences to *Bacteroidetes*, *Actinobacteria*, *Proteobacteria*, *Verrucomicrobia*, and *Firmicutes*. The authors identified five genetic loci involved in salt tolerance within the gut microbiota. More specifically, the genes identified were homologues of galE, mazG, and murB from three different species of the genus *Collinsella*, *Akkermansia*, and *Eggerthella*. Interestingly, one clone, assigned to the *Collinsella* genus, harbored these three genes; therefore, this is within 40 kb of metagenomic DNA. This is consistent with the co-localization of functionally related proteins frequently observed in prokaryotic genomes. Later, the same group performed transposition mutagenesis on one of the clones previously identified [34] and showed that transposon insertion in a gene eliminated the growth advantage under osmotic stress [35]. They named this locus stlA (for “salt tolerance locus A”) and predicted that it encodes a protein of 257 amino acids, which was totally novel, i.e., no similarity to any non-human associated metagenome nor any of the bacteria, eukaryotic, archaeal, viral, or plasmid genomes or sequences. Furthermore, they cloned the sltA gene in *E. coli* and showed that stlA conferred a salt tolerance phenotype. Another clone, previously identified by Culligan and colleagues in 2012, was further characterized by the same group using Phenotype MicroArray osmolyte plates [36]. It was shown that this clone was positive for the utilization/transport of L-carnitine in the presence of 6% NaCl. Sequencing and functional annotation did not reveal any genes related to known L-carnitine transport. After cloning the identified gene in *E. coli*, they showed that the sdtR gene did not confer the L-carnitine-associated phenotype but did confer an increased salt tolerance phenotype. Finally, in another work, Culligan et al. identified the locus brpA that encodes for a membrane protein with homology to a brp/blh-family *β*-carotene monooxygenase (the clone originated from the same initial screening in 2012) [37]. The cloning of the brpA gene in *E. coli* conferred a significant salt tolerance phenotype. Once again, this group identified a novel salt tolerance gene for the intestinal metagenome using functional metagenomics. To summarize these four papers from this group, they identified six genes (galE, murB, mazG, stlA, sdtR, and brpA) that confer salt tolerance. These are incredible examples of the strengths of functional metagenomics in deciphering food–microbiota interactions. 

Using the same type of samples, i.e., fecal samples, Tass et al. performed an extensive functional metagenomics study in 2011 to assess the human gut metagenome that encodes the carbohydrate-active enzymes (CAZymes) involved in dietary fiber breakdown [38]. CAZymes are involved in the breakdown of glycosidic linkage, and only a small number of glycoside hydrolases are encoded by the human genome, which limits our ability to digest dietary carbohydrates. Sequence-based metagenomic studies revealed the major role of the human gut microbiota in the metabolism of dietary carbohydrates since more than 150 families of CAZymes were highlighted. To explore their functional diversity and to assess their potential in the catabolism of dietary fibers, Tass and colleagues performed a high-throughput screening of 156,000 clones from a fecal sample of a vegetarian subject. The metagenomic library represented 5.42 Gb of metagenomic DNA, and each clone was bearing DNA fragments ranging from 30 to 40 kb. First, the 156,000 clones were screened for their ability to hydrolyze beta-glucan, xylan, beta-(1-4)-galactan, pectin, and amylose on a solid plate. From this first stage, they obtained 310 positive clones that were further screened for their capacity to degrade various polysaccharide structures and to work at high temperatures and extreme pH. At the end of the refined second screening, 26 positive clones were prioritized. The following step consisted of pyrosequencing and analyzing the sequences of the 26 clones. Data showed that they identified 73 CAZymes-encoding genes that resulted in 86 modules assigned to 35 known CAZymes families. They also identified nine novel glycoside–hydrolase (GH) families involved in the catabolism of complex glycans. This study demonstrated the great interest in the functional metagenomic approach in terms of discovering enzymes in the metagenome. The same year, researchers from our laboratory also used functional metagenomics to target the CAZymes involved in the catabolism of specific carbohydrates, specifically in relation to *β*-d-glucuronidase activity [39]. In this study, our laboratory used three fosmidic metagenomic libraries consisting of bacterial DNA extracted from the feces of healthy donors and patients with inflammatory bowel disease but also from a colorectal cancer patient biopsy obtained from the healthy distal part of the ileum. From these libraries, they screened 6136 clones for their activity on p-nitrophenyl-*β*-d-glucuronide substrate and on an agar plate supplemented with 5-bromo-4-chloro-3-indolyl-*β*-d-glucuronide. They identified 19 positive clones, from which 15 were assigned to *Firmicutes*. Moreover, one clone exhibited strong *β*-D-glucuronidase activity, and the gene encoded a *β*-D-glucuronidase that had different features compared to known *β*-D-glucuronidases (i.e., distant amino acid sequence homologies and an additional C terminus domain). Later, Patrascu and colleagues, from our laboratory, used functional metagenomics to decipher the ability of ileal mucosa-associated microbiota to degrade plant cell wall polysaccharides from dietary fibers [40]. They pointed out the fact that the digestion function of the dietary fibers was attributed to the microorganism of the distal part of the gastrointestinal tract, and most studies have focused on this part. In this study, they created a library of 20,000 clones from the ileal mucosa-associated microbiota of humans comprising *E. coli* recombinant fosmid clones with inserts ranging from 30 to 40 kb. The screening was performed on solid plates for carboxymethylcellulase, xylanase, *β*-glucanase, and lichenase activities. Out of the library, five clones displayed xylanase activity, and six clones produced both endoglucanase and xylanase activities. For the second screening, the 11 clones were degrading mixed-linkage polysaccharides (such as *β*-1,3-1,4-glucan) and lichenan, and none of them displayed xyloglucanase activity. The sequencing of the 11 clones and the sequence analysis revealed a broad range of CAZymes encoding genes from *Bacteroides* and *Clostridia* species but also Polysaccharide Utilization Loci. The study demonstrated the presence of bacterial genomes that encode a diversity of CAZymes in the ileum. Therefore, the ileal mucosa-associated microbiota has a specific function in relation to plant cell wall polysaccharide degradation. Functional metagenomics has once again proved its relevance in the discovery of enzymes in the metagenome. 

For several years, humans have consumed supplemented food to enrich their intestinal microbiota. Among them, prebiotics support the growth and activity of health-promoting bacteria. They are composed of oligosaccharides and polysaccharides that are non-digestible by the host. An interesting functional metagenomics study was conducted in 2013 by Cecchini et al. to decipher the metabolization of prebiotics by the gut microbiota [41]. In this study, they used 20,000 clones of two metagenomic libraries originating from the ileal mucosa and fecal microbiota. They screened their capacity to grow on a solid plate supplemented with xylo-oligosaccharides, lactulose, and fructo-oligosaccharides. They identified positive clones from the fecal and ileal libraries and subsequently performed a second screening step to validate and discriminate the prebiotic oligosaccharide hydrolysis ability of the positive clones. The clones were able to release monosaccharides from media containing xylo-oligosaccharides, fructo-oligosaccharides, and lactulose. The clones selected through the use of fructo-oligosaccharides were also able to degrade inulin. Altogether, they showed that uncultivated bacteria of the colon and the ileum were well equipped for the metabolization of prebiotics. Interestingly, sequence similarities in the clones were observed for species such as *Bifidobacterium*, *Bacteroides*, *Faecalibacterium*, *Streptococcus* and *Eubacterium*. Here, functional metagenomics was successfully used to discover bacteria that possess the enzymatic machinery to hydrolyze prebiotic carbohydrates. 

### 2.3. Functional Metagenomics of Microbiota–Host Interactions

Apart from its major contribution to the digestion of certain dietary constituents, the intestinal microbiota has another important function, as follows: crosstalk with the host through epithelial cells. Indeed, the intestinal microbiota is in direct contact with a mucosal barrier (composed of host-derived glycans) that lines the host epithelium. The mechanisms underlying the crosstalk between the host and its microbiota remain poorly understood, and, therefore, different strategies have been developed to unravel the impact of microorganisms on the regulation of signaling pathways and cell proliferation. 

A significant number of studies have been conducted on cultivable bacterial strains (aerobic and anaerobic) to study the bacteria–host interactions and successfully demonstrated the impact of the microorganisms on several molecular mechanisms. For example, Nepelska and colleagues from our laboratory selected 57 representative commensal bacterial strains and studied their impact on the modulation of a key nuclear receptor in colonocytes, PPARγ, which links metabolism and inflammation to the microbiota [42]. They showed that two strains, *Atopobium* and *Prevotella*, were able to up-regulate two PPARγ-targeted genes (ANGPTL4 and ADRP) that are involved in the phosphorylation of PPARγ via ERK1/2. In another study, O’Cuív et al. showed that two *Bacteroides vulgatus* strains were able to activate the NF-κB (an important transcription factor of epithelial cells that controls the inflammatory response and mediates the response to environmental stimuli) pathway in a strain- and growth-phase-specific manner [43]. To assess the immunomodulatory activities of the *Bacteroides vulgatus* culture extract, we used the enterocyte-like reporter cell line HT-29/Kb-seap-25 NF-κB. In a similar manner, we used the HT-29/Kb-luc-E reporter system to study the anti-inflammatory effect of several *Streptococcus salivarius* and *Streptococcus vestibularis* strains [44]. We showed that the strains’ supernatants were able to down-regulate NF-κB activation and that two strains were able to produce a metabolite that down-regulates the secretion of interleukine-8. These are some examples of successful studies that were conducted to unravel the impact of easily cultured bacterial strains on the modulation of host response. However, as mentioned previously, most of the microorganisms that constitute our intestinal microbiota are not cultivable, and conventional studies are not relevant to assessing their impact on the host. Therefore, functional metagenomics appears to be the technique of choice to discover the function of non-cultivable bacterial strains and unknown genes on host physiology. 

To overcome the limitations of culture-dependent techniques, Gloux and colleagues performed a functional screening of metagenomic libraries in 2007 to identify candidate genes involved in epithelial cell growth modulation [45]. Herein, we used two metagenomic libraries from the pooled fecal microbiota of six healthy subjects and six patients in remission from CD that gave access to 565 clones. These clones were screened for the growth modulation of HT-29 human intestinal epithelial and CV1 African green monkey kidney fibroblast cell lines by using crystal violet staining. We were able to identify 59 clones that modulate the proliferation of the cells. Interestingly, the clones were taxonomically assigned in majority to uncultured bacteria and, for the rest, to the main gut phyla *Bacteroidetes*, *Firmicutes*, *Proteobacteria*, and *Actinobacteria*. Moreover, the authors used transposon mutagenesis and subcloning on five clones to identify the genes responsible for the modulation of these cell line proliferations. The candidate genes identified encoded for several ATP-binding cassette (ABC) systems, a glutamate synthase subunit, a specific 16S rRNA gene and a RecD gene homologue. Further in the study, they adapted the method to screen, in a high-throughput manner, 20,160 clones from the mucosal microbial fraction of the ileum of a healthy individual. The screening was based on the quantification of intracellular ATP via luminescence as a cell growth indicator of HT-29 cells. From this step, they identified nine clones that show inhibition activities in terms of HT-29 cell growth. This study was one of the first approaches developed to quantify the modulation of cell growth through the use of metagenomic clones, and it successfully showed the possibilities that this approach offered. 

Later, in 2010, our group developed a high-throughput cell-based screening assay that enabled the detection of NF-κB pathway modulation in intestinal epithelial cells [46]. In this study, 2640 clones were screened, each bearing a 40 kb insert, from the intestinal microbiota of Crohn’s disease (CD) patients on stably transfected NF-κB-reporting HT-29 cells. From the clone lysates tested, 171 were able to modulate the NF-κB signaling pathway, with 22 that enhanced the reporter system activity and 149 that down-regulated it. One clone was selected for further study, and we identified a putative lipoprotein with an unknown function and an ABC transport system that belongs to the LolD family of lipoprotein transporters. Sequence analysis also revealed that the metagenomic insert originated from a new *Bacteroides* strain, which was recently assigned to *Bacteroides vulgatus* [43]. This study reinforced the potential of functional metagenomics in identifying the genes involved in microbiota–host crosstalk. Following our approach, another group also used functional metagenomics to assess the modulation of NF-κB transcription factor. Indeed, Cohen et al. developed high-throughput fluorescent microscopy screening to identify bacterial effector genes that activate NF-κB [47]. In this work, they screened three metagenomic libraries, 75,003 clones issued from stools from ulcerative colitis patients, CD patients and healthy donors using HEK293 cells stably transfected with NF-κB GFP reporter plasmid. Following the screening, they identified 143 clones that were able to reproducibly activate GFP expression in HEK293:NF-κB:GFP cells. Further transposon mutagenesis and sequencing allowed for the identification of 26 commensal bacteria effector genes that are predicted to encode proteins with diverse ligand-binding, anabolic, and catabolic functions. Interestingly, one effector gene issued from the three libraries and was found to encode genes involved in the production of N-acyl-3-hydroxypalmitoyl-glycine that activates the G protein-coupled receptor GPCR132/G2A, which mediates immune functions. More recently, in 2019, the same group also used HEK293:NF-κB:GFP cells to screen a so-called multigenomic library for NF-κB-inducing activity [48]. In this study, a multigenomic cosmid library was constructed from the purified genomic DNA of 116 human-associated bacterial strains (from stool, anterior nares, buccal mucosa, posterior fornix, supraglottic plaque, and tongue dorsum) and comprised a total of 13,300 clones. Filter-sterilized culture broth from individual clones was applied to HEK293:NF-κB:GFP cells, and NF-κB activation was measured as the ratio of live-GFP expressing cells to total live cells using a High Content Microscope. Among the clones tested, 21 clones significantly induced GFP expression and, therefore, NF-κB activation. From these 21 clones, they identified 17 unique genomic regions that arise from the major taxa of the multigenomic library. Furthermore, they performed transposon mutagenesis and sequencing on seven clones to identify the genes responsible for the activity of the NF-κB transcription factor. The termed “microbiome bacteria effector genes” were predicted to encode proteins with diverse LPS core biosynthesis, membrane transporters, cell wall hydrolases and unknown functions. These studies showed the power of functional metagenomics in terms of identifying bacterial effector genes from human microbiota and providing tools to unravel the mechanisms through which commensal bacteria interact with the host and, notably, in these examples, with the NF-κB transcriptional factor. 

The host epithelium is composed, among other cell types, of goblet cells that secrete mucin glycans, which are the interface between the microbiota and the host and act as a protective barrier. This rich environment in glycans offers colonization advantages for microbial species that possess the ability to bind and degrade mucus. In the previous part (Section 2.2), we have shown that gut bacteria are equipped with a large repertoire of CAZymes that support the host’s digestion of dietary fibers. Moreover, culture-based studies have shown that intestinal microorganisms are also able to use the glycans that are associated with human glycoproteins as a source of energy. Laville et al. performed a functional metagenomics study to identify the metabolic pathways involved in the degradation of glycans that compose the mucus in uncultured bacteria [49]. In this study, the authors screened a library of 20,000 clones from the ileum mucosa microbiota using several chromogenic substrates. Thus, they identified 124 clones that degrade host sialidases, *β*-D-N-acetyl-glucosaminidase, *β*-D-N-acetyl-galactosaminidase, and *β*-D-mannosidase. The authors selected clones with complementary glycosidase activity and with the highest level of activity to perform a second screening. It consisted of testing the 13 clones on human intestinal mucus using lectin-binding experiments. In this way, they highlighted nine clones with the ability to degrade the glycan structure. Moreover, they sequenced 13 clones to identify the mucin utilization loci and showed that they were assigned to *Bacteroides vulgatus*, *Bacteroides massiliensis*, *Bacteroides plebeius*, *Bacteroides uniformis*, and *Faecalibacterium prausnitzii*. The genes identified were predicted to encode proteins involved in the sensing of glycans, the breakdown of glycan structures into oligosaccharides, and in the central carbohydrate metabolism (mainly sialic acid metabolism). It was the first study to highlight glycan-mediated interactions between the host and its microbiota in humans. However, a previous functional metagenomics study was conducted to assess the adherence capacity of the microorganisms of the murine intestinal microbiota [50]. The authors screened a library of 5472 clones originating from the large-bowel microbiota of mice for their biofilm-forming capability (using crystal violet staining). They identified two clones that had 4.8- and 7.5-fold more robust biofilm formation than the control on solid glass. Further in vivo experiments showed that both exhibited more vigorous adherence to the host epithelium and were able to colonize the mouse intestine. Following transposon mutagenesis, sequencing and sequence analysis, they suggested that the sequences are derived from *Bacteroides* species. The genes identified were predicted to encode lysozyme-like and competence proteins. Both of these studies showed the relevance of functional metagenomics in deciphering the mechanisms employed by uncultured bacteria to degrade host mucus and adhere to the intestinal epithelium. These two functions may give certain microorganisms of the intestinal microbiota colonization advantages. 

### 2.4. Hot-Spots in Functional Metagenomics

Functional metagenomics is a relatively recent approach, but, as we have seen, it was successfully employed in several studies to decipher the food–microbiota and microbiota–host interactions. Several teams developed various functional-based screenings that enable the study of a wide range of mechanisms. Nowadays, and in the future, the challenge is to develop relevant techniques to elucidate unknown functions of the microbiota through the use of automated high-content screening, microfluidics, and solid plate experiments. For example, very recently, Tauzin and colleagues employed an ultra-high-throughput metagenomic approach based on droplet microfluidics [51]. Herein, a library of metagenomic clones (19,501 clones in *E. coli*) was encapsulated in droplets and screened for *β*-N-acetylgalactosaminidase activity. To assess enzymatic turnover in droplets, a fluorogenic glycoside analog was added to the cells, and they were sorted using FACS. To recover hit clones, the droplets were de-emulsified and plated onto agar. Then, a secondary screening was performed on 2232 clones for their hydrolytic activity. They sequenced 22 different clones and identified several CAZymes, carbohydrate esterases, and polysaccharide lyases that were assigned to the *Bacteroides* genus. With this study, the authors demonstrated the relevance and speed of the method. This method can be applied to all types of metagenomic libraries and allows for the screening of thousands of clones in one hour of work. This is a great example of recent developments in the field of functional metagenomics. 

One of the hottest topics in the field of functional metagenomics is the development of other species as host bacteria for metagenomic inserts. While *E. coli* is largely used in functional metagenomics studies, it has some limitations in the expression of genes from other Gram-negative species and from Gram-positive species. For Gram-negative species, Lam et al. developed a *Bacteroides* system in 2018 to screen DNA from the intestinal microbiota [52]. As *Bacteroides* is an abundant genus of the intestinal microbiota, they proposed that the use of *Bacteroides thetaiotaomicron* will be relevant and will favor the expression of *Bacteroides* genes at the transcriptional level and, therefore, increase hit rates from screening. They confirmed that a fosmid of approximately 45 kb can be successfully conjugated into *Bacteroides thetaiotaomicron*, but they also highlighted that homologous recombination can arise in the host genome. As mentioned earlier, the use of host Gram-positive bacteria will give access to specific signals of Gram-positive bacteria that will enhance the heterologous expression of genes for this Gram. It is expected that the use of Gram-positive bacteria as host will allow for the enhanced secretion of proteins via specific signal peptides and will promote the exposure of bioactive proteins and, once again, increase hit rates from screening. To date, only a few studies have been performed with Gram-positive hosts to screen metagenomic libraries. For example, Dobrijevic and colleagues used a *Bacillus subtilis* system for the presentation of proteins from Gram + bacteria [53]. Additionally, McMahon et al. developed functional screening of metagenomic libraries in *Streptomyces lividans*, showing that clones were functional in this species [54]. While the metagenomic library originated from soil, they have demonstrated the relevance of using a different host than *E. coli*.

Our laboratory has developed several tools to screen (meta)genomic libraries, bacteria, and their metabolites across a wide range of cellular functions and pathways (Table 1). For example, works have been published on the effect of gut commensal bacteria on AhR [55], Transforming growth factor beta (TGF-*β*) [56], PPARγ [42], and Indoleamine 2,3-dioxygenase-1 (IDO1) [57]. However, we are still working on the development of complementary screening tools to target other signalization pathways such as Activator protein 1 (AP1), Heat Shock Transcription Factor 1 (HSF1), or calcium mobilization and the expression of genes of interest like Muc-2, IL-10, Thymic Stromal Lymphopoietin (TSLP), or Angiopoietin-like 4 (AngPTL4). This will offer a wilder panel of targets and allow a better understanding of the crosstalk between the gut microbiota and the host.

However, this methodology has its limitations. It requires high-throughput approaches and, therefore, expensive robotization and is therefore not accessible to everyone. In recent years, there has been growing interest in tissue microbiota with very low biomass, heavily contaminated with human DNA. This severely limits the functional metagenomics approach since the vast majority of clones screened carry human DNA. Finally, functional metagenomics studies deal with a very large amount of data and therefore require particular attention in terms of statistical analysis. A robust data treatment must be incorporated into the pipeline to increase the sensitivity of the analysis and the success of the study, as reported by de Wouters et al., from our group [58]. The implementation of gene databases will allow the precise identification of the genes highlighted in studies. Future development in data analysis may also include artificial intelligence that will facilitate and accelerate the analysis of large sets of data and images (from microscopy, for example). 

**Table 1:** Tools developed to screen (meta)genomic libraries, bacteria, and their metabolites in terms of several cellular functions and pathways. References refer to publications linking targets of interest and gut microbiota.

Reporter Cell Lines	Target	References
Caco-2-AhR-Luc	AhR	[55]
Colo-205-IL-10-Luc	IL-10	[59]
HCT116-HSF1-GFP	Heat shock factor 1	[60]
HEK293:NF-κB:GFP	NF-kB	[47,48]
Hela-HSF1-GFP	Heat shock factor 1	[60]
Hela-LC3-GFP	Autophagosome marker LC3	[61]
HT-29- TGF*β*1-Luc	TGF*β*1	[56]
HT-29-AhR-Luc	AhR	[55]
HT-29-AP1-Luc	AP1	[62]
HT-29-CCND1-Luc	Cyclin D1	[63]
HT-29-HSF1-GFP	Heat shock factor 1	[60]
HT-29-IDO-1-Luc	IDO-1	[57]
HT-29-Muc2-LUC	Muc2	[64]
HT-29-NF-kB-SEAP	NF-kB	[46]
HT-29-PPARγ-Luc	PPARγ	[42]
HT-29-TSLP-Luc	TSLP	[65]
SW1116-ANGPTL4-luc	AngPTL4	[58]

## 3. Conclusions

Based on the studies that we described here and our developed experience over 15 years, we have revealed that functional metagenomics is a powerful technique in terms of discovering signaling pathways and functions in the crosstalk between food compounds, the gut microbiota, and the host. The creation of metagenomic libraries originating from different organisms (mice, cows, and humans) and the use of specific screening systems have permitted the discovery of new *β*-glucanase, hydrolytic, and carbo-hydrate-active enzymes as well as genes encoding for ABC systems, glutamate synthase subunits, putative lipoproteins, proteins with diverse LPS core biosynthesis, membrane transporters, and cell wall hydrolase. The assignation of these genes to specific species or genera provided precious information on the capacity of the members of the microbiota in terms of performing important functions, such as the degradation of plant cell wall polysaccharides, the hydrolysis of bile salt, the modulation of epithelial cell growth, the degradation of human glycan, adherence to host cells and the modulation of the immune system. We believe this approach is fundamental to discovering and deciphering new functions of the intestinal microbiota and, of course, other ecosystems of the human body. However, this approach requires permanent improvements of the screening systems, the management of large amounts of data, and the development of new host species for metagenomic inserts in order to unravel yet-unknown functions.
